# Comparison of depression and anxiety symptom networks in reporters and non-reporters of lifetime trauma in two samples of differing severity

**DOI:** 10.1016/j.jadr.2021.100201

**Published:** 2021-12

**Authors:** Alicia J. Peel, Chérie Armour, Joshua E.J. Buckman, Jonathan R.I. Coleman, Susannah C.B. Curzons, Molly R. Davies, Christopher Hübel, Ian Jones, Gursharan Kalsi, Monika McAtarsney-Kovacs, Andrew M. McIntosh, Dina Monssen, Jessica Mundy, Christopher Rayner, Henry C. Rogers, Megan Skelton, Abigail ter Kuile, Katherine N. Thompson, Gerome Breen, Andrea Danese, Thalia C. Eley

**Affiliations:** a Social, Genetic and Developmental Psychiatry Centre; Institute of Psychiatry, Psychology & Neuroscience; King's College London, London SE5 8AF, UK; b School of Psychology, Queens University Belfast, Belfast BT7 1NN, Northern Ireland; c Centre for Outcomes Research and Effectiveness (CORE), Research Department of Clinical, Educational & Health Psychology, University College London, London WC1E 7HB, UK; d iCope – Camden and Islington Psychological Therapies Services, Camden & Islington NHS Foundation Trust, 4 St Pancras Way, London NW1 0PE, UK; e UK National Institute for Health Research (NIHR) Biomedical Research Centre, South London and Maudsley NHS Trust, London SE5 8AF, UK; f Department of Medical Epidemiology and Biostatistics, Karolinska Institutet, Stockholm, Sweden; g National Centre for Mental Health, MRC Centre for Neuropsychiatric Genetics and Genomics, Cardiff University, Cardiff CF24 4HQ, UK; h Division of Psychiatry, University of Edinburgh, Edinburgh EH10 5HF, UK; i Department of Child & Adolescent Psychiatry, Institute of Psychiatry, Psychology & Neuroscience, King's College London, London SE5 8AF, UK; j National and Specialist CAMHS Trauma, Anxiety, and Depression Clinic, South London and Maudsley NHS Foundation Trust, London SE5 8AF, UK

**Keywords:** Trauma, Depression, Anxiety, Network analysis, Self-report

## Abstract

**Background:**

Reported trauma is associated with differences in the course and outcomes of depression and anxiety. However, no research has explored the association between reported trauma and patterns of clinically relevant symptoms of both depression and anxiety.

**Methods:**

We used network analysis to investigate associations between reported trauma and depression and anxiety symptom interactions in affected individuals from the Genetic Links to Anxiety and Depression (GLAD) Study (n = 17720), and population volunteers from the UK Biobank (n = 11120). Participants with current moderate symptoms of depression or anxiety were grouped into reporters and non-reporters of lifetime trauma. Networks of 16 depression and anxiety symptoms in the two groups were compared using the network comparison test.

**Results:**

In the GLAD Study, networks of reporters and non-reporters of lifetime trauma did not differ on any metric. In the UK Biobank, the symptom network of reporters had significantly greater density (7.80) than the network of non-reporters (7.05).

**Limitations:**

The data collected in the GLAD Study and the UK Biobank are self-reported with validated or semi-validated questionnaires.

**Conclusions:**

Reported lifetime trauma was associated with stronger interactions between symptoms of depression and anxiety in population volunteers. Differences between reporters and non-reporters may not be observed in individuals with severe depression and/or anxiety due to limited variance in the presentation of disorder.

## 1 Introduction

Depression and anxiety are among the most common mental health disorders worldwide, each with an estimated lifetime prevalence of 30% (Bandelow & Michaelis, 2015; Kessler et al., 2012; McManus et al., 2016). Depression and anxiety disorders are highly comorbid, particularly in primary care settings (Hirschfeld, 2001; Saha et al., 2021). It is estimated that half of adults with one disorder have symptoms of the other (McManus et al., 2009). Depression and anxiety share many risk factors, including cognitive biases (Naragon-Gainey, 2010), environmental exposures (Kessler, 1997) and genetic influences (Purves et al., 2019; Waszczuk et al., 2014). However, they are highly heterogeneous, meaning that individuals with the same diagnosis display very different patterns of symptoms and trajectories of illness (Karstoft et al., 2020; Milaneschi et al., 2016; van Loo et al., 2018). Knowledge of how specific risk factors influence heterogeneity is limited, but such an understanding may provide insight into the mechanisms that underlie these disorders, enabling treatments targeted to certain risk pathways.

A key environmental risk factor for both depression and anxiety is lifetime trauma, namely events that threaten injury, loss of life, or physical integrity (APA, 2013). Around 55-80% of individuals with clinical depression or anxiety report experiencing traumatic events (Davis et al., 2020; Hepgul et al., 2016; Mazure, 1998; Miloyan et al., 2018). Reporting traumatic events is associated with earlier age of onset, chronicity of disorder, greater symptom severity, poorer treatment outcomes and increased risk of relapse and recurrence (Buckman et al., 2018; Francis et al., 2012; Hovens et al., 2012; Moitra et al., 2011; Nanni et al., 2012; Nelson et al., 2017; Wiersma et al., 2009). Furthermore, subjective self-reports of trauma have been associated with a greater risk of later psychopathology than objective records (Danese & Widom, 2020). This indicates that the subjective experience of trauma may have a greater influence on the development and presentation of disorder than the occurrence of the event. It is possible that heterogeneity in depressive and anxiety disorders may be influenced by the subjective experience or interpretation of these exposures, as captured by self-reports (Fried et al., 2015; Keller et al., 2007). Despite this, limited research has explored associations between reported trauma and differences in patterns of depressive symptoms, and even less has considered symptoms of anxiety (Muscatell et al., 2009).

Using network analysis, it is possible to model associations between risk factors, such as reported trauma, and patterns of symptoms (Boschloo et al., 2015). Network theory conceptualises disorders as dynamic structures of interacting and mutually reinforcing symptoms (van Borkulo et al., 2017). This enables investigation of the arrangement and strength of the associations between symptoms, and allows their differential importance, or centrality, to be assessed (Armour et al., 2017).

Only one recent study has explored differences in depressive symptom networks associated with reported trauma (van Loo et al., 2018). The sample of over 5000 Han Chinese females with severe, recurrent depression was grouped into those who did and did not report any lifetime trauma, including childhood abuse and neglect, death of a relative, serious accident/illness, assault or sexual abuse. Symptom networks were indistinguishable between participants who did and did not report lifetime trauma, with no statistically significant differences in structure or connectivity (van Loo et al., 2018). This was also seen for other genetic and environmental risk factors. It was proposed that the severity of the sample may have caused a ceiling effect, whereby limited variation in the presentation of depression resulted in minimal differences in symptom associations (Terluin et al., 2016). If a ceiling effect did contribute to these findings, samples with more diverse severity may show greater variation between groups (van Loo et al., 2018). Furthermore, despite the high comorbidity between depression and anxiety, only one symptom of anxiety was included (“Nervous, jittery, anxious”).

It is essential to explore how these findings generalise to samples that include a broader range of symptom severity and presentation. Researching associations between risk factors and clinical features of depression and anxiety is necessary for optimising more targeted care, whereby treatments are tailored to specific risk factors or presentations. Knowledge of whether differences in symptoms networks are associated with reported trauma in representative samples has the potential to inform treatment strategies and guide provision of healthcare resources.

This study had two aims. First, to compare current depression and anxiety symptom networks between reporters and non-reporters of lifetime trauma, in order to assess differences in symptom interactions associated with reporting lifetime trauma. Second, to assess the impact of sample severity by conducting analyses in two samples, a study of individuals with lifetime depression or anxiety and a sample of population volunteers who were not ascertained on the basis of having any disorder, referred to as ‘unselected volunteers’. If ceiling effects are present in severe samples, we would expect that no differences would be identified between reporters and non-reporters in the sample of individuals with lifetime disorder.

## 2 Methods

### 2.1 Study design

We utilised two UK cohorts, the Genetic Links to Anxiety and Depression (GLAD) Study and the UK Biobank. The GLAD Study is a cohort of individuals aged 16+ with lifetime experience of depression and/or anxiety, launched in 2018 (Davies et al., 2019). At the time of these analyses, 30524 participants had completed the sign-up questionnaire. In the GLAD Study, 86.7% of participants reach diagnostic criteria for major depression, and 64.5% meet criteria for an anxiety disorder (Davies et al., 2019). The GLAD Study includes participants with relatively severe depression and/or anxiety, with high rates of recurrence, functional impairment and comorbidity (Davies et al., 2019). Almost all (96.1%) report having received treatment (Davies et al., 2019). Compared to the general population, participants are younger, more female, less ethnically diverse and spend more years in education (Davies et al., 2019).

The UK Biobank is a cohort of ~500000 unselected population volunteers, aged 40-70 recruited between 2007-2010 (Sudlow et al., 2015). In 2017, 157366 participants completed the follow-up Mental Health Questionnaire (Davis et al., 2020). In the UK Biobank, 24% of participants meet criteria for a lifetime diagnosis of major depression and 7% for generalised anxiety disorder (Davis et al., 2020). These rates are slightly higher than prevalence estimates from the Health Survey for England (2014), which found that 19% of adults report a lifetime diagnosis of depression, and 6% report a lifetime diagnosis of generalised anxiety disorder (Bridges, 2015). The measures in the GLAD Study and the UK Biobank Mental Health Questionnaire overlap substantially, facilitating research combining these cohorts (Davies et al., 2019). Compared to the general population, participants are older, spend more years in education, have higher socio-economic status and fewer health conditions (Davis et al., 2020).

### 2.2 Measures

#### 2.2.1 Depression and anxiety symptoms

Current depressive and anxiety symptoms were assessed in order to minimise recall bias. Depressive symptoms were assessed using the Patient Health Questionnaire (PHQ-9) (Kroenke & Spitzer, 2002), a widely used self-report measure that assesses the nine DSM-5 (Diagnostic and Statistical Manual of Mental Disorders) symptoms of major depressive disorder (APA, 2013). Symptoms of anxiety were assessed using the Generalised Anxiety Disorder scale (GAD-7) (Spitzer et al., 2006), a self-report measure that assesses the seven DSM-5 symptoms of generalised anxiety disorder. On both scales, participants report how frequently they have been bothered by each symptom over the last two weeks on a four-point Likert item from ‘Not at all’ (0) to ‘Nearly every day’ (3), resulting in a maximum total symptom score of 48. The use of both scales together as a composite measure of depression and anxiety has shown good internal reliability, convergent and construct validity in clinical samples (Kroenke et al., 2016). Total scores on the composite measure of 10, 20, and 30 indicated mild, moderate, and severe levels of depression/anxiety, respectively (Kroenke et al., 2016).

#### 2.2.2 Reported trauma

Reported trauma was assessed using a measure created by the UK Biobank Mental Health steering group (Davis et al., 2020). This included the Childhood Trauma Screener (Grabe et al., 2012), a short form of the Childhood Trauma Questionnaire (Bernstein, 1998) that has been validated in clinical samples with acceptable internal consistency (α = .757; Grabe et al., 2012). A screener capturing the adult equivalents of these items was designed based on questions from the National Crime Survey (Davis et al., 2020; Khalifeh et al., 2015). Additionally, participants completed a six-item checklist, asking about experiences of events commonly associated with post-traumatic stress disorder (Frissa et al., 2016).

In order to identify exposures most related to our outcomes, we selected the seven items previously associated with major depression in the UK Biobank (Table 1; Coleman et al. 2020): childhood emotional abuse and neglect, childhood sexual abuse, domestic emotional, physical and sexual abuse, and sexual assault. To assess group differences, a dichotomous variable indicating presence or absence of reported lifetime trauma was created. Reporters of trauma were defined as any participant who reported one or more of the seven lifetime traumas. Participants reporting none were defined as non-reporters. Those with incomplete data were excluded. Details of this measures and response coding according to published cut-offs is given in the Supplementary Material.

**Table 1:** Reported trauma questionnaire items.

Form of trauma	Questionnaire item
Childhood emotional neglect	When I was growing up… I felt loved
Childhood emotional abuse	When I was growing up… I felt that someone in my family hated me
Childhood sexual abuse	When I was growing up… Someone molested me (sexually)
Domestic physical abuse	Since I was sixteen… A partner or ex-partner deliberately hit me or used violence in any other way
Domestic emotional abuse	Since I was sixteen… A partner or ex-partner repeatedly belittled me to the extent that I felt worthless
Domestic sexual abuse	Since I was sixteen… A partner or ex-partner sexually interfered with me, or forced me to have sex against my wishes
Sexual assault	In your life, have you… Been a victim of a sexual assault, whether by a stranger or someone you knew

### 2.3 Participants

Participants who met criteria for clinically relevant levels of current depression or anxiety symptoms at the time of participation were included in these analyses. This was defined as a score ≥ 10 on either the PHQ-9 or GAD-7, the threshold for moderate symptoms (Kroenke & Spitzer, 2002). Of the 30524 GLAD study participants who had completed the study prior to 27th October 2019, 19575 (64%) participants had complete symptom data and met criteria for current symptoms of depression or anxiety. We excluded 1855 (9%) participants with missing reported trauma data, resulting in 17720 participants being included. Of the 157366 UK Biobank participants who completed the Mental Health Questionnaire, 12168 (8%) participants had complete symptom data and met criteria for current symptoms of depression or anxiety. We excluded 1048 (9%) participants with missing data on reported trauma, resulting in 11120 participants being included.

### 2.4 Statistical analyses

#### 2.4.1 Network estimation

Symptom networks consisting of the nine depressive symptoms from the PHQ-9 and seven anxiety symptoms from the GAD-7 were estimated in reporters and non-reporters. Pre-processing checks assessing collinearity were conducted using the ‘goldbricker’ function from the R package ‘networktools’, which compares correlations in order to identify nodes which are likely to measure the same underlying construct. Collinearity was not identified so all 16 symptoms were included in the networks. The Gaussian Graphical Model was used for network estimation with polychoric correlations (Epskamp, 2016), with edges indicating partial correlations (Epskamp & Fried, 2018). The ‘graphical least absolute shrinkage and selection operator’ (glasso) (Tibshirani, 1996), a form of regularisation, is utilised within the estimation of the Gaussian Graphical Model to limit the number of spurious edges (Epskamp et al., 2018) controlled by minimising the Extended Bayesian Information Criterion (EBIC) (Chen & Chen, 2008). All analyses were performed in R version 3.6.0 using R package bootnet 1.2.2 (Epskamp et al., 2018), which incorporates functions from the package qgraph 1.6.2 (Epskamp et al., 2012).

Networks were presented using the qgraph package (Epskamp et al., 2012), with blue edges representing positive associations and red edges negative. Thickness and darkness of the edge indicates strength of the association, where thicker, darker edges are stronger. Networks are arranged using a version of the Fruchterman-Reingold algorithm (“spring” layout in qgraph) (Fruchterman & Reingold, 1991), where highly connected nodes are placed closer together.

The recommended three-step bootstrapping procedures were followed for each of the four networks, to assess edge weight accuracy and centrality stability (Epskamp et al., 2018). The full procedure is detailed in the Supplementary Material.

#### 2.4.2 Network comparison

To investigate differences between reporters and non-reporters, networks were statistically compared using the network comparison test, a permutation test which randomly rearranges group membership and refits the two network models 10000 times, applying Holm-Bonferroni correction for multiple testing (van Borkulo et al., 2017). More information about this test and the metrics compared is given in the Supplementary Material. Firstly, networks were compared on symptom centrality, a measure of which symptoms are most influential in the networks, using two local centrality indices (Epskamp & Fried, 2018). Strength is the sum of absolute edge weights connected to each node. Expected influence is the sum of edge weights retaining the positive or negative value of the association. Expected influence gives greater weight to symptoms with more positive associations, and less to those with negative associations, in order to identify symptoms that may make the most logical clinical targets (Robinaugh et al., 2016). Secondly, network connectivity was compared using three key metrics. Global network strength (S), the absolute sum of all edge weights, is an indicator of how densely connected the overall network is. Network structure is a measure of the difference in weight between the two networks for the edge that most greatly differs (M), indicating variation in the arrangement of symptom associations. If network structure significantly differs, differences in edge strength are tested (E), to assess which specific edge weights differ across the two networks. Network comparison was performed using R package NetworkComparisonTest 2.0.1 (37).

### 2.5 Sensitivity analyses

To assess the impact of unequal sized groups, sensitivity analyses with equal sized subsets of reporters and non-reporters were conducted, reported in the Supplementary Material.

Sensitivity analyses were also conducted to explore differences associated with the developmental timing and type of the reported trauma. Symptom networks estimated in reporters of childhood trauma, reporters of adulthood trauma, reporters of emotional trauma and reporters of physical/sexual trauma were compared to the networks of non-reporters, using the network comparison test as outlined above.

## 3 Results

### 3.1 Sample characteristics

The demographic and clinical characteristics of the full samples and comparison groups are shown in Table 2. Of the 17720 GLAD Study participants, 3756 (21%) were non-reporters and 13964 (79%) were reporters of trauma. Of the 11120 UK Biobank participants, 4321 (39%) were non-reporters and 6799 (61%) were reporters of trauma.

**Table 2:** Characteristics of the Genetics Links to Anxiety and Depression (GLAD) Study and the UK Biobank.

	Full sample	Non-reporters of trauma	Reporters of trauma	Difference between non-reporters and reporters
GLAD Study, N	17720	3756 (21%)	13964 (79%)	
Age (years)	36.0 (SD = 13.7)	35.7 (SD = 14.2)	36.1 (SD = 13.6)	t(5741.9) = -1.481, p = .14, d = .03
Sex (female)	14301 (81%)	2547 (68%)	11754 (84%)	χ^2^(1) = 507.8, p < .001, V = .17*
Current depression (PHQ-9 ≥ 10)	15981 (90%)	3159 (84%)	12822 (92%)	χ^2^(1) = 198.3, p < .001, V = .10*
Current anxiety (GAD-7 ≥ 10)	12191 (69%)	2474 (66%)	9717 (70%)	χ^2^(1) = 18.89, p < .001, V = .03*
Total symptom score	27.8 (SD = 8.8)	25.6 (SD = 8.1)	28.4 (SD = 8.9)	t(6369.7) = -18.172, p < .001, d = .32*
PHQ-9 score	15.5 (SD = 5.4)	14.0 (SD = 5.2)	15.9 (SD = 5.4)	t(6049) = -19.646, p < .001, d = .36*
GAD-7 score	12.3 (SD = 5.2)	11.6 (SD = 5.2)	12.5 (SD = 5.2)	t(5978.7) = -9.102, p < .001, d = .17*
UK Biobank, N	11120	4321 (39%)	6799 (61%)	
Age (years)	53.1 (SD = 7.8)	53.8 (SD = 7.9)	52.6 (SD = 7.6)	t(8864.3) = 7.7094, p < .001, d = .15*
Sex (female)	7165 (64%)	2353 (55%)	4812 (71%)	χ^2^(1) = 306.3, p < .001, V = .17*
Current depression (PHQ-9 ≥ 10)	8332 (75%)	2992 (69%)	5340 (79%)	χ^2^(1) = 121.1, p < .001, V = .10*
Current anxiety (GAD-7 ≥ 10)	6176 (56%)	2412 (56%)	3764 (55%)	χ^2^(1) = 0.2075, p = .649, V = .01
Total symptom score	21.8 (SD = 7.5)	20.5 (SD = 6.7)	22.6 (SD = 7.8)	t(10195) = -14.673, p < .001, d = .28*
PHQ-9 score	11.9 (SD = 5.2)	10.9 (SD = 5.0)	12.5 (SD = 5.3)	t(9508.3) = -16.277, p < .001, d = .31*
GAD-7 score	9.9 (SD = 5.2)	9.7 (SD = 5.0)	10.1 (SD = 5.2)	t(9437.1) = -4.2855, p < .001, d = .08*

* = p-values < .005 (Bonferroni-corrected), PHQ-9 = Patient Health Questionnaire, GAD-7 = Generalised Anxiety Disorder scale, d = Cohen's d, V = Cramer's V, SD = standard deviation, N = sample size, t = Student's t-test, χ2 = Chi-squared

Non-reporters and reporters in the GLAD Study significantly differed in sex, current depression, current anxiety and total symptom score (the frequency of reported PHQ-9 and GAD-7 symptoms). Non-reporters and reporters in the UK Biobank significantly differed in age, sex, current depression and total symptom score. The largest differences between non-reporters and reporters were in sex (GLAD: non-reporters 68% female, reporters 84% female, UK Biobank: non-reporters 55% female, reporters 71% female) and total symptom score (GLAD: non-reporters 25.6, reporters 28.4, UK Biobank: non-reporters 20.5, reporters 22.6). Overall, in both samples, reporters were more likely to be female, to meet criteria for current depression, and to have higher total symptom scores than non-reporters.

### 3.2 Symptom networks

**Fig. 1:**
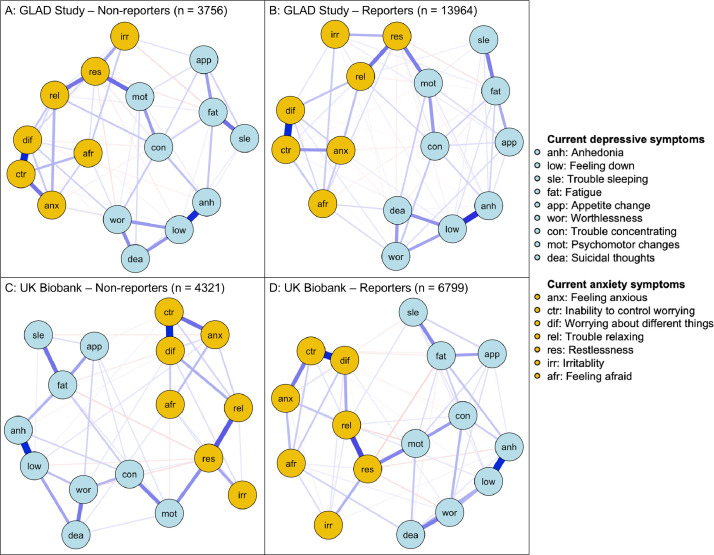
Networks of 16 depression and anxiety symptoms in Genetic Links to Anxiety and Depression (GLAD) Study non-reporters (1A; n = 3756) and reporters of trauma (1B; n = 13964) and in UK Biobank non-reporters (1C; n = 4321) and reporters of trauma (1D; n = 6799). Blue edges indicate positive associations, red edges indicate negative associations. The thickness and brightness of the edge indicates the strength of the association, with thicker and darker edges representing stronger associations. The layout of the networks is based on the Fruchterman-Reingold algorithm, that arranges nodes with a greater number of strong connections closer together and nodes with greater centrality near the centre.

Fig. 1 shows the networks of 16 depression and anxiety symptoms for non-reporters and reporters of trauma in the GLAD Study and the UK Biobank. Analyses of accuracy and stability are given in the Supplementary Material.

### 3.3 Comparison of symptom networks

#### 3.3.1 Symptom centrality

For all networks, case-dropping bootstraps indicated high stability for both centrality measures (CS-coefficients = 0.75).

**Fig. 2:**
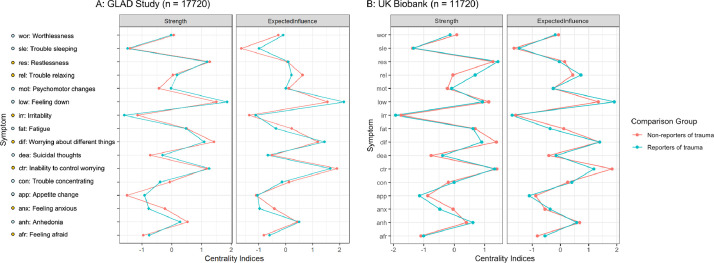
Comparison of strength and expected influence of the depression and anxiety symptom networks for non-reporters and reporters of trauma in the Genetic Links to Anxiety and Depression (GLAD) Study (2A, N = 17720) and the UK Biobank (2B, N = 11120). Centrality indices are displayed using standardised z-scores to aid interpretation.

Low mood, inability to control worrying, worrying about different things, and restlessness were the most central symptoms for all groups in terms of strength, indicating that they had the greatest absolute sum of connecting associations (Fig. 2). In terms of expected influence, in which the direction of associations is retained, restlessness was not among the most central symptoms, indicating that it shared a combination of positive and negative associations.

In both samples, the network comparison test revealed that the centrality of all of the 16 symptoms did not differ significantly between reporters and non-reporters of trauma for either measure (p > .05).

#### 3.3.2 Network connectivity

For all networks, bootstrapping tests for accuracy revealed narrow confidence intervals around edge weight estimates, indicating good precision.

For the GLAD Study, in non-reporters, 59 out of the maximum 120 symptom associations, edges, were estimated to be non-zero, compared to 70 in reporters. For the UK Biobank, in non-reporters, 48 edges were estimated to be non-zero, compared to 59 in reporters.

In all networks, the strongest edges were between low mood and anhedonia (edge weight = 0.56-0.61), and between inability to control worry and worrying about different things (0.62-0.67).

**Fig. 3:**
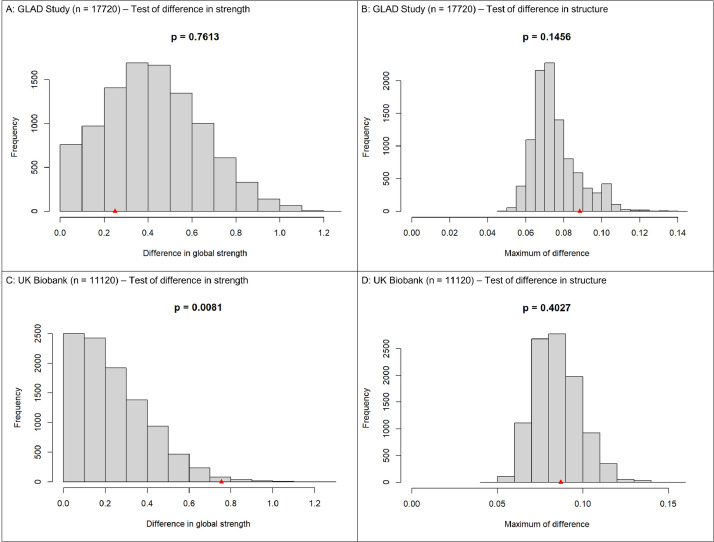
Results of permutation testing for network comparisons of global strength and network structure in the Genetic Links to Anxiety and Depression (GLAD) Study (N = 17720; 3A and 3B) and the UK Biobank (N = 11120; 3C and 3D). Panels 3A and 3C represent the distribution of differences in global network strength (S). Panels 3B and 3D represent the distribution of the maximum difference in edge weights (M). For all figures, p = the proportion of the 10000 randomly allocated permutation groups with a difference at least as large as the difference in the original networks estimated in non-reporters and reporters. The red triangle on the x-axis indicates the difference between the original two networks.

The results of the network comparison test for network connectivity are given in Fig. 3. The red triangle on the x-axis indicates the observed difference between the networks derived from non-reporters and reporters of trauma. In the GLAD Study, the network comparison test revealed that the networks of non-reporters and reporters did not significantly differ in global strength (S) (7.79 compared to 8.04, S = 0.25, p = .761) or network structure, based upon the maximum difference of any edge weight (M) (M = .09, p = .145). In contrast, in the UK Biobank, the networks of non-reporters and reporters did significantly differ in global strength (7.05 compared to 7.80, S = 0.75, p = .008), with the network of reporters having a greater total strength of associations. The networks did not differ significantly in network structure (M = .09, p = .403), indicating that the symptoms were arranged similarly in two groups despite differences in strength.

### 3.4 Sensitivity analyses

Sensitivity analyses assessing differences associated with the developmental timing and type of the reported trauma are presented in full in the Supplementary Material. Results broadly mirror those of the main analyses. In the GLAD Study, the networks of reporters of childhood trauma, reporters of adulthood trauma, reporters of emotional trauma and reporters of physical/sexual trauma did not significantly differ from the network of non-reporters. In UK Biobank, the networks of reporters of adulthood trauma (7.76 compared to 7.05, S = 0.71, p = .014), reporters of emotional trauma (7.05 compared to 7.78, S = 0.73, p = .011) and reporters of physical/sexual trauma (7.05 compared to 7.73, S = 0.68, p = .017) had significantly greater total strength of associations than the network of non-reporters. The network of reporters of childhood trauma did not significantly differ from the network of non-reporters.

## 4 Discussion

In the GLAD Study, networks of current depression and anxiety symptoms did not differ between reporters and non-reporters of lifetime trauma on any metric. In the UK Biobank, the network of reporters was more densely connected than that of non-reporters, with connectivity similar to that seen in the GLAD Study.

The findings from the GLAD Study replicate an earlier investigation of differences in depressive symptom networks in a sample of females with severe, recurrent depression (van Loo et al., 2018). We extended existing research by including male participants, and by exploring DSM symptoms of both depression and anxiety. The GLAD Study is a relatively severe sample, with very high rates of chronicity, recurrence, comorbidity and treatment receipt (Davies et al., 2019). Our findings support the hypothesis that once a certain severity of disorder is present, reported trauma is not associated with meaningful differences in depression and anxiety symptom associations (van Loo et al., 2018).

In the UK Biobank, we found a different pattern of results. The network of reporters of trauma had significantly greater global strength than the network of non-reporters, indicating a more densely connected network. This result was also observed in sensitivity analyses when non-reporters were compared to reporters of adulthood trauma, emotional trauma and physical/sexual trauma, but was not seen for reporters of childhood trauma. The meaning of network density is currently debated (McElroy et al., 2019). Overall, past research is indicative of an association between network density and persistence of symptoms in clinical groups, although the mechanisms that underlie this relationship are poorly understood (McElroy et al., 2019; Schweren et al., 2018; van Borkulo et al., 2015). The density of the network of reporters in the UK Biobank (7.80) was similar to that observed in both groups in the GLAD Study (7.79 and 8.04). This implies that, compared to those who do not report trauma, reporters from the UK Biobank display a pattern of symptoms that is more reflective of the complex or persistent depression and anxiety experienced by GLAD Study participants. This reflects the literature displaying associations between reported trauma and greater symptom severity and chronicity or recurrence (Francis et al., 2012; Hovens et al., 2012; Moitra et al., 2011; Nanni et al., 2012; Nelson et al., 2017; Wiersma et al., 2009).

The differences between the findings in the GLAD Study and the UK Biobank could reflect systematic differences between these cohorts. The UK Biobank is a population-based volunteer sample of older adults, whereas GLAD Study participants are recruited based on a lifetime history of depression or anxiety, and are on average younger. However, these differences are also in line with the explanation that less severe samples may display larger differences between groups, possible due to greater variance and dimensionality (Fried, van Borkulo, et al., 2016; van Loo et al., 2018). As well as having lower prevalence of lifetime depression and anxiety overall, participants from the UK Biobank displayed lower current total symptom scores. Both reporters and non-reporters in UK Biobank displayed moderate symptoms, with total symptom scores of 22.6 and 20.5, respectively, whereas reporters and non-reporters from the GLAD Study displayed moderate-severe symptoms, with total scores of 28.4 and 25.6. It is possible that these differences in severity between the two cohorts contributed to greater ability to identify differences between reporters and non-reporters in the UK Biobank.

Despite some differences in density, the structures of all four networks were strikingly similar. Low mood, inability to control worrying and worrying about different things were the most central symptoms for reporters and non-reporters in both samples. Low mood and inability to control worrying are considered to be cardinal symptoms for depression and generalised anxiety, respectively, and are used in brief two-item screening measures (Löwe et al., 2005; Plummer et al., 2016). These symptoms are consistently identified among the most central network symptoms of depression in adults (Fried et al. 2016; Hakulinen et al. 2020; Beard et al. 2016) and across development in children and adolescents (McElroy et al., 2018). The consistent centrality of these core symptoms is a reassuring indicator of their use as screening items and reiterates their importance as therapeutic targets (Beard et al., 2016). Furthermore, the arrangement of the associations between the symptoms of depression and anxiety reflects robust findings in the literature, with many connections between the two disorders (Beard et al., 2016; Fried, Epskamp, et al., 2016; McElroy et al., 2018). The replicability of these network properties across numerous cohorts of differing ages, gender balance and severity levels emphasises the robust overlap between symptoms of depression and anxiety (Fried, 2015). These results indicate that the activation of symptoms is likely to transfer between disorders highlighting the importance of considering both sets of symptoms in research and clinical contexts (Fried, Epskamp, et al., 2016).

These findings should be considered in light of some limitations. The data collected in the GLAD Study and the UK Biobank are self-reported with validated or semi-validated questionnaires. Therefore, the associations may reflect common method variance. The participants in the current study were all experiencing current depression or anxiety symptoms, which may have biased reports of past traumatic events. Retrospective reports of trauma also show poor agreement with prospectively collected accounts (Baldwin et al., 2019). Poor agreement between retrospective and prospective accounts of trauma does not necessarily signify poor validity, but indicates that these measures identify largely different groups of individuals (Baldwin et al., 2019), suggesting that the findings of the current study may only be relevant to individuals who retrospectively self-report trauma. Nevertheless, retrospective reports of trauma are linked to a greater risk of psychopathology than objective accounts, indicating that the subjective experience plays an important role in the development of disorder (Danese, 2020; Danese & Widom, 2020). This emphasises the importance of investigating the outcomes associated with retrospectively reported trauma (Danese, 2020). Finally, recruitment in both cohorts is biased towards those who spend more years in education and have higher socio-economic status. Therefore, it is unclear how these findings would apply to the general population, particularly as higher socio-economic status is associated with lower likelihood of experiencing trauma (Brattström et al., 2015).

A key strength of these analyses was the inclusion of symptoms of both depression and anxiety, as these disorders are highly comorbid. The PHQ-9 and GAD-7 do have some limitations, in that various symptoms related to sleep and appetite changes, for example poor appetite and overeating, are combined into single questions, and not every symptom that would meet DSM criteria is included, for example weight loss. However, in using these concise measures, a comprehensive collection of DSM-relevant symptoms from the last two weeks could be assessed, limiting recall bias. Furthermore, the criteria of moderate symptoms used to identify participants were based on total scores, and did not require the endorsement of specific symptoms. This meant that endorsement rates for core symptoms were not artificially inflated in the analytic sample, and did not need to be excluded from analyses, as they have been in previous studies (Kendler et al., 2018; van Loo et al., 2018). However, combining two measures that are psychometrically distinct with high internal consistency may have increased associations with core symptoms, as they are likely to be highly correlated with other items from the same measure, but not with items from the other measure. Nevertheless, these symptoms have also been identified as the most central in studies using one measure incorporating both depressive and anxious symptoms (Fried, Epskamp, et al., 2016; McElroy et al., 2018). Finally, it is important to reiterate that this study sought only to identify those with current moderate depression or anxiety symptoms, rather than cases of major depression and/or generalized anxiety disorder.

These findings have several implications. Primarily, they suggest that reported trauma may be associated with a pattern of symptoms consistent with more severe or complex depression and anxiety, and therefore that individuals who report trauma may benefit from a greater duration or intensity of treatment. They also indicate that once a certain severity of disorder is met, reported trauma is not associated with variation in symptomatic presentation. This implies that for those with severe presentation, the specific risk pathways that underlie the disorder may not be useful in differentiating between those who are and are not likely to experience poor outcomes. However, reported trauma may be a more relevant risk factor for identifying those at greater risk of chronicity or poor treatment response in individuals with less severe presentation. As discussed, further research is needed to unpick the relationships between density and persistence of disorder, especially as network analysis becomes an increasingly popular tool in psychiatric research. This includes investigation of how network density relates to chronic depression and response to treatments in large, clinical samples. The current study provides evidence for the importance of utilising samples that display variance in symptom severity when exploring differences in networks between subgroups.

Finally, this study speaks to the recent call for increased efforts to assess the replicability and generalisability of network models (Robinaugh et al., 2020), and highlights the increasing consistencies identified between network models of depression and anxiety. Given that symptoms of both disorders were among the most central, and connectivity existed across symptoms of depression and anxiety, these results indicate that transdiagnostic treatment approaches that target both depressive and anxious symptoms may be advantageous for widespread symptom improvement (Newby et al., 2015). Further replication attempts are required to strengthen confidence in the findings of network analysis studies. Future research should focus on investigating how the concept of centrality can be applied to clinical practice, whether targeting the most central symptoms leads to greater overall improvement in presentation, and whether participants with certain symptom interactions respond differently to various therapeutic strategies.

## 5 Conclusions

Reported trauma was not associated with meaningful differences in depression and anxiety symptom networks in a sample of severely affected individuals, but was associated with greater network density in a sample of population volunteers. The density of the network of the reporters from the UK Biobank was comparable to both networks estimated in GLAD Study participants. These findings suggest that reported trauma may be associated with a pattern of symptoms consistent with more severe or complex depression and anxiety. Further research exploring the mechanisms underlying network density is necessary, and it may be important to conduct such research in samples with greater variance in the presentation of disorder.

## Authors' contributions

AJP, TCE, and AD designed the study. SCBC, MRD, GK, MMK, DM, HCR and KNT contributed to acquisition of data. AJP carried out the statistical analysis. JRIC, CH and KNT provided input in the analysis. All authors assisted with interpretation of the findings and critically reviewed the manuscript. AJP revised the manuscript for final submission. All authors read and approved the final manuscript.

## Funding

UK Biobank was established by the Wellcome Trust, Medical Research Council, Department of Health, Scottish Government and Northwest Regional Development Agency. This research has been conducted using the UK Biobank Resource, under the application 16577. The GLAD Study is supported by the 10.13039/501100012618National Institute of Health Research (10.13039/100017751NIHR) 10.13039/100017751BioResource, NIHR Biomedical Research Centre [IS-BRC-1215-20018], HSC R&D Division, Public Health Agency [COM/5516/18], MRC Mental Health Data Pathfinder Award (MC_PC_17,217), and the National Centre for Mental Health funding through 10.13039/100012068Health and Care Research Wales. TCE is part funded by a program grant from the UK Medical Research Council (MR/V012878/1). GB is part funded by a program grant from the UK Medical Research Council (MR/M021475/1). AD is part funded by the National Institute for Health Research (NIHR) Biomedical Research Centre at South London and 10.13039/100009362Maudsley NHS Foundation Trust and 10.13039/100009360King's College London part-funded by a program grant from the UK Medical Research Council (MR/M021475/1). AJP is supported by an ESRC studentship. JEJB was funded by the 10.13039/100010269Wellcome Trust (201292/Z/16/Z). CR is supported by a grant from Fondation Peters to TCE and GB. MS is funded by an NIHR Maudsley Biomedical Research Centre studentship.

## Data availability

Supplementary material, R code and adjacency matrices from this analysis are available at: https://osf.io/zda8s/

The data that support the findings of this study are available to approved researchers, subject to registration and application process conducted by the GLAD Study and UK Biobank.

## Declaration of Competing Interest

GB has received honoraria, research or conference grants and consulting fees from Illumina and Otsuka. AMM has received research support from Eli Lilly, Janssen, and the Sackler Foundation, and has also received speaker fees from Illumina and Janssen. The remaining authors declare no conflict of interests.
